# Numerical simulation study on the evolution law of mechanical properties of different metamorphic coals after heat treatment

**DOI:** 10.1038/s41598-024-59051-1

**Published:** 2024-04-08

**Authors:** Xun Zhang, Hongyu Lai, Ge Huang, Bing Lu, Huimin Liang

**Affiliations:** 1 College of Mining, Liaoning Technical University, Fuxin, 123000 Liaoning China; 2 Institute of Safety Engineering and Technology, Liaoning Technical University, Fuxin, 123000 Liaoning China; 3 College of Safety Science and Engineering, Liaoning Technical University, Fuxin, 123000 Liaoning China

**Keywords:** Discrete element, Mechanical properties, Hot cracking, Numerical simulation, Coal spontaneous combustion, Coal, Engineering

## Abstract

In order to study the effect of temperature on the structure and mechanical properties of coal with different metamorphic degree. Three coal samples with varying degrees of metamorphism were chosen for analysis. The discrete element software PFC2D is used to simulate the heat treatment and compression of coal. The findings indicate that during the heating process, low-order coal exhibits noticeable thermal cracks at an early stage, while thermal crack development in middle-order coal is concentrated in the later stages. In contrast, high-order coal demonstrates a more stable macroscopic structure. The strength and stiffness of low rank coal show the lowest value and decrease significantly within 135 °C. However, the strength and stiffness of medium rank coal decrease significantly after 135 °C. The changes of mechanical properties and damage modes of coal caused by thermal damage are often ignored, which may lead to the deviation of design and research results from the actual situation. Therefore, this study is of great significance to the prevention and control of coal mine disasters.

## Introduction

Coal is classified as a unique form of organic rock that possesses both fissures and pores, rendering it a double porous medium^1^. In the compound disaster mine of gas outburst and coal spontaneous combustion, the pressure relief and extraction of coal seam can realize outburst elimination. However, it is easy to cause the development of coal cracks, increase the intensity of air leakage, and then oxidize the coal body and induce coal spontaneous combustion^2,3^. To address the damage resulting from spontaneous coal combustion in mines, an investigation is conducted into the microstructural evolution law of coal seams under temperature influence^4^. Numerical investigation of the gas and temperature evolution during the process of spontaneous burning of coal in large hearths has been documented^5^, The occurrence of spontaneous coal combustion in coal mine operations continues to be a significant safety concern due to the intricate chemical and physical characteristics of coal^6,7^, The occurrence of spontaneous combustion in coal can result in detrimental effects on the coal body, leading to modifications in its physical characteristics^8^. Numerous scholars, both domestic and international, have conducted a multitude of studies on this particular subject matter. By studying the effect of high temperature on the organization and mechanical properties of hard coal, Su et al.^9^ found that the modulus of elasticity and uniaxial compressive strength of the coal samples decreased with the increase of heating temperature. Qinghe Niu et al.^10^, by studying the evolution and formation mechanism of closed pores in coal, found that the permeability of the coal body increases with increasing temperature, and thermal rupture plays a major role in the generation of pore cleavage. Wang et al.^11^ studying coal thermal expansion and mechanical property tests, it was found that both coal strength and stiffness showed a decreasing trend with increasing temperature. Zhai et al.^1^ by using low-field nuclear magnetic resonance (NMR) technology to study the changes in coal pore structure, it is found that the high-temperature and high-pressure NMR analyzer can simulate the environment of deep in-situ coal seams, and realize the real-time analysis and imaging of coal cores in the processes of hydraulic fracturing, gas injection and substitution, gas adsorption and desorption, and stress deformation. He et al.^12^ study of desorption characteristics of adsorbed gas in coal under uniaxial stress-temperature; The combined effect of pore pressure and temperature on the permeability of anthracite coal was studied by Yin^13^; Wan et al.^14^ to study the evolution of the elastic modulus of coal bodies under high-temperature triaxial stress, but the traditional tests have inherent limitations such as non-repeatability, inability to directly observe the microcracks generated by the interaction of mineral particles, and inability to comprehensively characterize the fracture evolution of coal bodies under different temperature boundary conditions, Potyondy et al. believe that combining with particle modeling is the current trend and future direction^15^.

So some scholars used numerical simulation to study and demonstrate. For example, Someone analyzed several fire scenarios in underground roadways, taking into account the main environmental variables: airflow, temperature, oxygen, and pollutants. The fire dynamics simulator (FDS) models the five most representative scenarios. The behavior before and after fire load is determined^16^. Through the experimental and simulation study of the dynamic characteristics of the impact failure process of gas-bearing coal under loading stress and gas environment, Kong et al.^17^ found that there were significant differences in the influence of initial pore cracks on the strength of coal. The destructive mechanical behavior of granite at high temperature^18^, Wanne, T. S. et al.^19^ used the PFC2D program to develop a numerical model of granite with different mechanical parameters, and Kumari et al. investigated the mechanical behavior of granite under in-situ stress and temperature conditions^20^, which simulated the expansion and rupture of the center of granite due to heat. Numerical simulations by Tang et al. through a coupled thermal-water-mechanical damage model and its application to rock column stability analysis^21^, showed test behavior similar to that observed in the laboratory; Liang et al.^22^ found that the percentage of shear-type cracks gradually increased with the increase of the stress difference of the initial stress state through the particle flow analysis of the thermal rupture characteristics of granite with different stress states under the action of thermal coupling. Some scholars have used technology to measure more detailed data, and used acoustic emission technology to capture the deformation and failure process of coal samples^23^. CT scan is used to scan the internal structure of the specimen to study the influence of uniaxial stress on the structure of coal pore fracture system^24,25^. Today there are more experimental or simulation studies of anaerobic pyrolysis and thermal rupture of different components of rocks, and from this point of view, complete rock compression experiments cannot fully reveal the detailed rupture process, especially the type of rupture^26^.

Scholars have made some progress in the mechanical properties of coal by temperature through a large number of studies, and found that the degree of oxidation, the scope of oxidation caused changes in the mechanical properties of coal, and at the same time, high-temperature pyrolysis caused changes in the internal pores and cracks of coal body, permeability and thermal conductivity. However, most of these researches focus on the macroscopic evolution of the same kind of coal, and most of the researches on thermal damage and damage mechanics^27^ also focus on the rocks. Little consideration has been given to the effect of temperature on coals with different degrees of metamorphism, and complete rock compression experiments cannot fully reveal the detailed rupture processes, especially the type of rupture^26^, It can be analyzed more intuitively from the point of view of micromechanics, and there are few methods to study the micromechanics of coal specimens at high temperatures; moreover, there are significant differences in terms of temperature on the change of internal cracks and mechanical properties of coals with different degrees of metamorphism. Therefore, in order to further investigate the changes in the mechanical properties of coal with different degrees of metamorphism and the evolution of internal cracks during coal oxidation, this paper adopts the PFC2D particle flow software, and compares the experimental results of the previous experiments while^8,28,29^ and simulation experience^30^, the numerical simulations is completed.

## Basic particle flow theory and methods

### Basic theory of particle flow

The fundamental principles underlying the particle flow program are the force–displacement law^31^ and Newton's second law. The force–displacement law governs the manner in which the contacting components interact, whereas Newton's second law controls the relative position and contact relationship of the particles in contact with the wall. The motion equation is expressed by two sets of vector equations. The relationship between the force and the linear motion is another group that represents the resultant moment and the rotation. The relationship of motion is shown in formula (1) and formula (2) respectively.

1 $$F_{i} = m(\ddot{x} - g_{i} )$$

2 $$M_{i} = H_{i}$$

The formula (1) is a linear motion, and the formula (2) is a rotational motion. In the formula: *F*_*i*_ is the resultant force; *m* is the total mass of particles; *g*_*i*_ is the acceleration of gravity.

**Figure 1:**
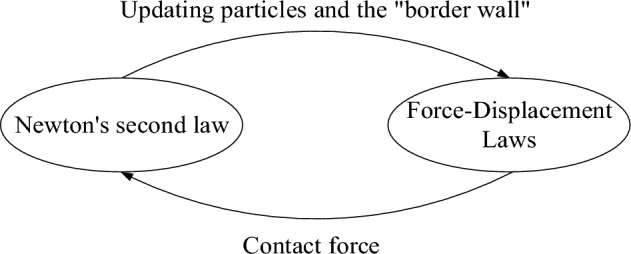
PFC Calculation principle.

The aforementioned laws are reiterated during the computational process until the computational model achieves a condition of equilibrium or experiences damage instability. As depicted in Fig. 1.

**Figure 2:**
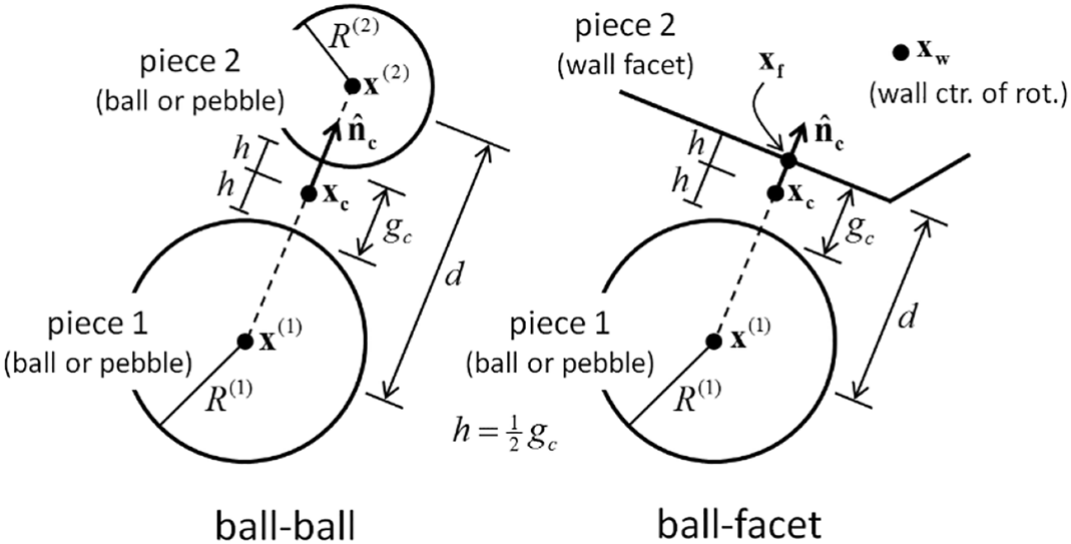
Contact plane positions and normal directions for two basic contact types: ball-ball and ball-surface.

The contact plane is positioned at the center of the interaction volume between the two objects, with its direction extending from object 1 towards object 2 and tangential to object 1. The contact plane's position (*x*_*c*_) and normal direction (${\widehat{n}}_{c}$) are provided in Fig. 2.

3 $$x_{c} = x^{\left( 1 \right)} + \left( {R^{\left( 1 \right)} + \frac{{g_{c} }}{2}} \right) \cdot \widehat{n}_{c}$$

4 $$\widehat{n}_{c} = \left\{ {\begin{array}{*{20}c} {\frac{{x^{\left( 2 \right)} - x^{\left( 1 \right)} }}{d},ball - ball} \\ {\frac{{x_{f} - x^{\left( 1 \right)} }}{d},ball - facet} \\ \end{array} } \right.$$

5 $$g_{c} = \left\{ {\begin{array}{*{20}c} {d - \left( {R^{\left( 1 \right)} + R^{\left( 2 \right)} } \right),ball - ball} \\ {d - R^{\left( 1 \right)} ,ball - facet} \\ \end{array} } \right.$$

6 $$d = \left\{ {\begin{array}{*{20}c} {\left\| {x^{\left( 2 \right)} - x^{\left( 1 \right)} } \right\|,ball - ball} \\ {\left\| {x_{f} - x^{\left( 1 \right)} } \right\|,ball - facet} \\ \end{array} } \right.$$

where *x*^(b)^ and *R*^(b)^ are the center and radius of ball (b), respectively; *x*_*f*_ is the point on the wall closest to *x*^(1)^; *x*_*w*_ is the center of rotation of the wall, and *g*_c_ is the contact gap. The contact gap refers to the minimal signed distance that exists between the surfaces of the workpiece. It should be noted that the contact gap is considered negative when the surfaces overlap.

### Particle flow contact constitutive model

The particle flow model does not provide direct determination of the macroscopic mechanical characteristics of the specimen. Instead, it requires the specification of geometric parameters of the particles, contact qualities, and appropriate fine-scale parameters in order to accurately represent the macroscopic mechanical properties of the specimen. Hence, it is crucial to undertake the necessary steps of choosing the particle contact intrinsic model and fine-scale parameter calibration in order to establish the PFC2D model. Additionally, it is imperative to introduce a fissure within the PFC2D model to successfully create a numerical representation of fissured coal samples.

**Figure 3:**
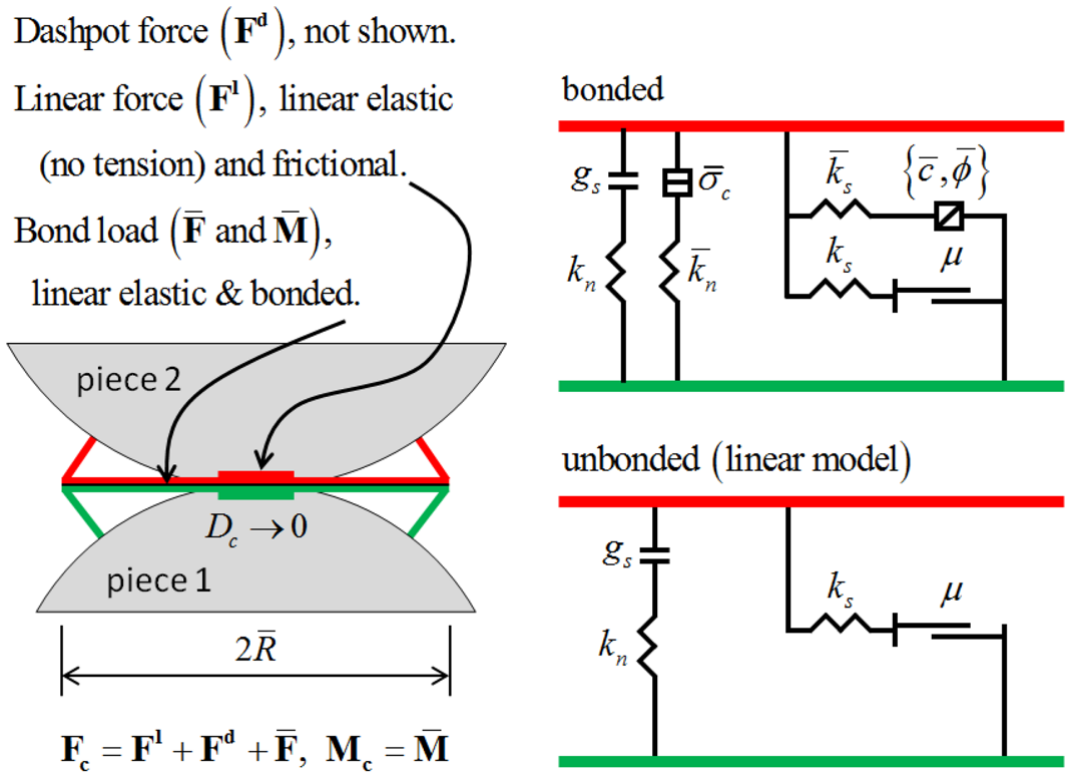
Parallel bonding model.

Particle flow programs consist of two separate types of particle-to-particle adhesion models, specifically the contact adhesion model and the parallel adhesion model. Within the framework of the contact adhesion paradigm, adhesion is exclusively detected at the minuscule contact region formed between two particles. This particular aspect functions as the link between neighboring particles. The particles exhibit a lack of resistance to rotational motion. The influence of contact stiffness persists on the particles as long as they maintain contact, even after the deterioration of the contact bond. The fundamental aim of the contact bond model is to facilitate the transmission of forces, and it is commonly utilized to describe bulk materials such as soils. On the other hand, the parallel bond concept entails the establishment of a link that acts within a confined circular cross-section between two particles. This relationship possesses the capacity to transmit both forces and moments. The decrease in stiffness is a result of bond rupture within the parallel bond model, as it is controlled by both the contact stiffness and the bond stiffness^32^. Once the connection is ruptured, the stiffness attributed to the bond is immediately eliminated. However, the stiffness connected with the contact between particles remains intact as long as the particles remain in touch. The application of numerical simulation methods has been widely seen in the examination of coal rock-like materials, specifically in the evaluation of their mechanical characteristics^33^, In this context, a parallel bond model has been developed to enhance the precise depiction of these substances in computational simulations. Figure 3, illustrates the attributes and rheological components of the linear parallel bond model.

## Principle of thermal coupling and thermal parameters

### Modeling and attribute assignment

Prior to conducting simulations, it is necessary to assign precise micromechanical parameters to the model. A significant number of numerical simulation tests are then performed, with a continuous process of adjusting the micromechanical parameters through a "trial and error method"^34^. The outcomes of the numerical simulations are subsequently compared with mechanical tests conducted in a controlled environment. This iterative process of comparison and adjustment continues until the numerical model aligns with the results of the mechanical tests. Once this consistency is achieved, the parameters of the numerical model can be confidently utilized in practical calculations^35^.

**Figure 4:**
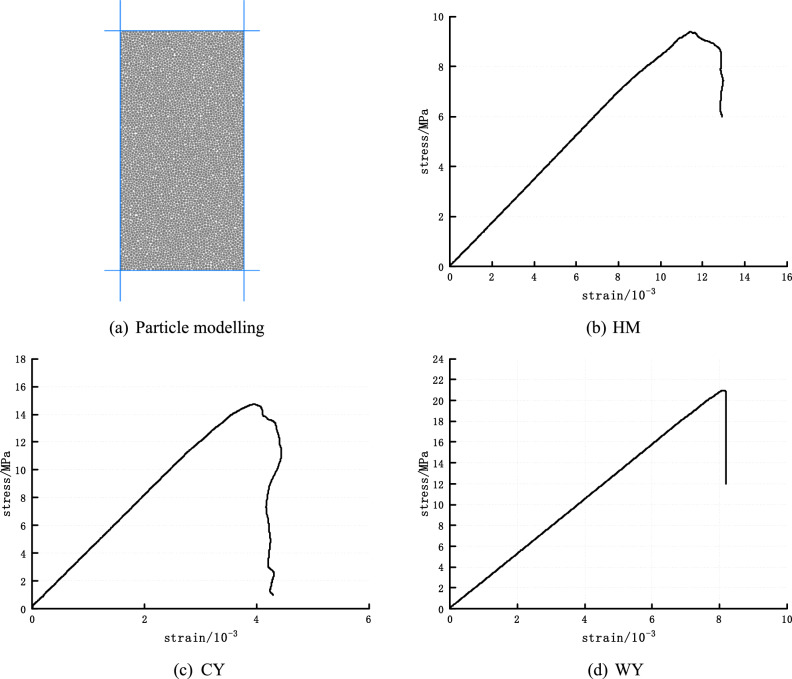
Uniaxial compression experiment based on PFC^2D^**.**

The numerical simulation utilized the parallel bonding model to conduct an indoor uniaxial compression test. Eight fine view parameters, namely emod, krat, pb_emod, pb_krat, pb_ten, pb_coh, pb_fa, and fric, were selected for this purpose. The Diameter of 50mm height of 100mm numerical model of coal samples was established with a density of 2500 kg/m^3^, a minimum particle radius of 0.4 mm. Considering the simulation accuracy and computational efficiency, the particle size ratio is set to 1.66, a uniform distribution of particle sizes, and a total number of particles of 4744, and the established PFC2D image^36^ is shown in Fig. 4.

Axial loading is implemented by subjecting the upper and lower walls to a consistent velocity, while the uniaxial compression test is conducted until the specimen undergoes failure and the load attains the predetermined threshold. The aforementioned parameters have been derived through iterative debugging of lignite (HM), long flame coal (CY), and anthracite (WY). Table 1 presents the relevant data. The generated uniaxial stress–strain curve is compared with the results obtained from prior indoor tests^8,28,29^.

**Table 1:** Model mechanical parameters.

	HM	CY	WY
emod/GPa	0.5	2.5	1.5
krat	2.5	3.7	2.5
pb_emod	0.5	2.5	1.5
pb_krat	2.5	3.7	2.5
pb_coh	2.5 × 10^6^	5.0 × 10^6^	8.0 × 10^6^
Pb_ten	6.0 × 10^6^	6.0 × 10^6^	9.0 × 10^6^
Pb_fa	30	30	30
fric	0.5	0.5	0.5

In Ref.^8^, the compressive strength of raw coal is 16.36 MPa, and the elastic modulus is 3.779GPa, which is similar to CY. In Reference^28^, the compressive strength of raw coal is about 8.5 MPa, and the elastic modulus is about 0.75Gpa, which is roughly the same as that of HM. In Ref.^29^, the compressive strength of raw coal is about 19 MPa, and the elastic modulus is about 2.5Gpa, which is similar to WY.

The nonlinearity of stress rise during uniaxial compression testing contrasts with the linearity shown in simulations. The presence of minuscule pores within the coal body results in a compaction phase during the initial loading stage. Furthermore, it is worth noting that the stiffness of the uniaxial compression test machine is insufficient. Additionally, it is a significant factor contributing to the nonlinearity shown in the stress–strain curve. Currently, there exists a lack of viable resolution to this issue. The numerical simulation results are depicted in Fig. 4. The simulated peak stress and strain exhibit congruence with the experimental findings, displaying comparable trends in their curves and the presence of macroscopic cracks. The parameter adjustment method is a widely employed technique in numerical simulations of particle movement. The accuracy of the numerical model can be attributed to its similar mechanical properties to those of the experimental specimens, as stated in Ref.^34^. The numerical model has the potential to be employed in future thermodynamic simulations.

### Simulation scheme

The numerical model is configured with a temperature condition corresponding to room temperature (25 °C). The boundary condition is established by considering the high temperature in proximity to the wall, and the heat transfer process is simulated using numerical methods. In accordance with the preceding investigation on the oxidation degree of CY, the temperature was incrementally elevated to 70 °C, 135 °C, 200 °C, and 265 °C, thereafter undergoing natural cooling to reach room temperature (25 °C), among other conditions. The current state of crack development, as well as the quantity and spread of shear and tensile cracks during the process of heat transfer, were documented. The thermal age command was employed to demonstrate the chronological order of fissures. Prefabricated crack damage treatment was incorporated into the mechanical model to mimic the influence of temperature-induced cracks on mechanical properties at various temperatures. Next, the numerical model was subjected to a simulation using the uniaxial compression test.

### Thermal module principle

The thermal module in PFC is founded upon the principles of the Fourier heat conduction law. This module has the capability to replicate the dynamic heat conduction, heat storage, and the resulting strain and force development in materials that consist of rigid body particles. The built-in parallel bond model of PFC incorporates thermal strain. Thermal strain is produced as a result of the expansion of the bond model between particles due to the application of heat. The given phrase is:

7 $$\Delta R=\alpha R\Delta {\rm T}$$

The formula represents the variation in radius, denoted as Δ*R*, resulting from the influence of temperature. The linear thermal expansion coefficient of particles is represented by *α*, while ∆*T* represents the change in temperature.

The linear expansion coefficient of parallel bonding in perfluorocarbon (PFC) is a characteristic property. The expansion deformation is solely oriented along the normal direction, and it is considered to exhibit isotropy. The parallel bonding model exhibits deformation or contraction in the perpendicular direction, leading to a modification in its overall length. The expression for the variation of the normal bonding force is as follows:

8 $$\Delta \overline{{F_{n} }} = - \overline{{k^{n} }} A\Delta U^{n} = - \overline{{k^{n} }} A\left( {\overline{\alpha } \overline{L} \Delta T} \right)$$

$-\overline{{k }^{n}}$ is the normal bonding stiffness; *A* is the cross-sectional area of contact;$\overline{\alpha }$ is the linear expansion coefficient of parallel bonding;$\overline{L }$ is the bonding strength; Δ*T* Temperature change.

The thermal fracture calculation involves the consideration of a thermal coupling effect, which results in alterations in force and displacement on rock structures subjected to thermal influences. Mechanical calculations are employed for the purpose of assessing potential damage to the particle contact bond.

### Thermophysical parameters

In the PFC2D software, once the mechanical numerical model has been successfully debugged, the next step involves utilizing the integrated fish language of PFC to incorporate heat calculations into the model. To initiate a heat source, the initial temperature values for the four walls were set. The Configthermal command was then enabled to facilitate the simulation of the heating process. A uniform heating approach was employed to simulate the heating process. The temperature is increased by 5 °C in each iteration, with a total of 1000 iterations, while maintaining a continuous process of heat transfer. The process of cooling is accomplished by adjusting the temperature of the wall heat source to match the ambient room temperature of 25 °C, and subsequently relying on natural heat transfer mechanisms until the heat source reaches equilibrium with the room temperature. Prior to doing thermal calculations, it is necessary to give thermodynamic microscopic properties to all particles. The thermodynamic characteristics of the model proposed in reference^37^ are presented in Table 2, encompassing key properties such as the thermal expansion coefficient, specific heat capacity, and thermal conductivity.

**Table 2:** Thermodynamic parameters.

	Thermal expansion coefficient/(m·K^−1^)	Specific heat capacity /J/(kg·K)	Thermal conductivity/(W/mK)
HM	2.5 × 10^−5^	1150	0.30
CY	2.8 × 10^−5^	1022	0.32
WY	3.0 × 10^−5^	1200	0.35

## Results and discussion

### Number of cracks and their propagation law

The mineral particles within coal rock exhibit a continuum-like behavior, wherein their deformation is constrained by the need to maintain continuity. Consequently, the thermal expansion system of these particles is unable to freely deform in response to temperature variations, resulting in the creation of boundaries between them. Consequently, the coal body experiences a form of stress known as thermal stress. The structure of coal is susceptible to damage when exposed to specific temperatures, resulting in thermal stress. This thermal stress surpasses the tensile stress yield strength between coal particles, leading to the creation of micro fractures and a subsequent reduction in coal strength^38^.

**Figure 5:**
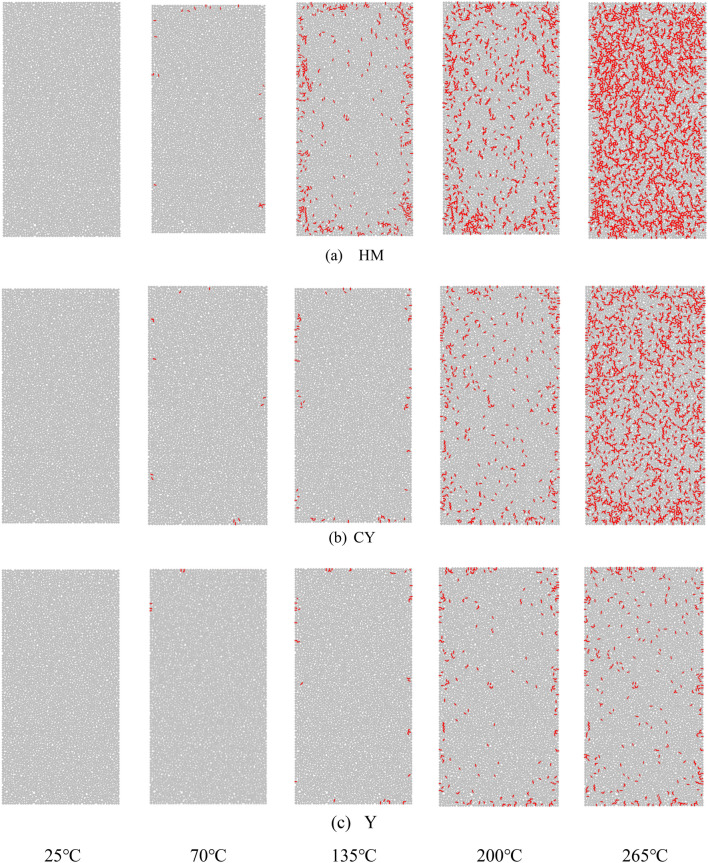
Crack distribution of coal after heat treatment.

As depicted in Fig. 5, the model exhibits a limited number of thermal cracks near its periphery when subjected to HM at a temperature of 70 °C. This indicates that the thermal stress has not yet surpassed the critical threshold for inducing cementation fracture in the coal body, thereby implying relative stability. However, at a higher temperature of 135 °C, the HM causes a significant escalation in the number of thermal cracks. Nevertheless, these cracks still predominantly manifest in the vici The phenomenon described can be attributed to the interaction between the initial gas and moisture present in the coal, resulting in volume expansion when subjected to heat and thermal stress generated by the coal body skeleton. This expansion leads to the formation of cracks within a short period of time, primarily due to uneven heat transfer and distribution at the surrounding edges. At temperatures of 200 to 265 °C, these cracks further increase, at a temperature of 200 °C, there are new cracks originating on the outer surface, the thermal expansion of the coal body and the surrounding column caused the cracks to propagate from the outside edges towards the core of the coal body. The initial fractures exhibited internal propagation, whereas the coal samples treated with CY at a temperature of 265 °C displayed a significant increase in the frequency of thermal cracks, both internally and outside. The WY material did not exhibit prominent thermal cracks under normal conditions. However, when subjected to temperatures ranging from 200 to 265 °C, it did form thermal cracks internally. Nevertheless, the total increase in the quantity of cracks generated was minimal.

The heating process results in notable variations in the macroscopic structures of the three forms of coal, owing to the varying degrees of coalification. The material denoted as HM exhibits the greatest quantity of thermal fractures among the studied materials. Additionally, it possesses the lowest temperature threshold for initiating thermal expansion, resulting in the formation of discernible thermal cracks. Secondly, it is worth noting that while CY also exhibits significant thermal expansion, the occurrence of thermal cracks is comparatively lower in CY as compared to HM. Additionally, the temperature threshold for the formation of such cracks in CY is higher than that in HM. In conclusion, the material denoted as WY had the lowest incidence of thermal cracks, demonstrating a notable level of stability even when subjected to a temperature rise of 6 degrees. No discernible fractures were noticed on the surface of the coal throughout the process of heating. The findings suggest a positive correlation between coal rank and resistance to thermal expansion, as well as a negative correlation between coal rank and the impact of temperature on the macroscopic structure.

### Stress–strain curve

The stress–strain curve and macroscopic failure of the simulation results are extremely similar to those of previous experiments^8,28,29^, which not only demonstrates the accuracy of previous experiments but also demonstrates the reliability of the numerical simulation.

**Figure 6:**
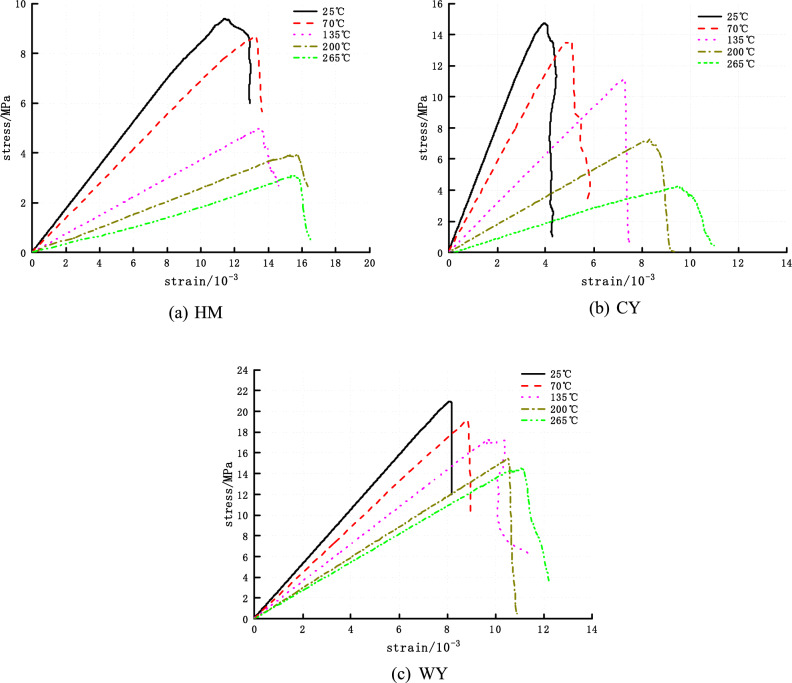
The stress–strain curves of three kinds of coal after heat treatment.

The stress–strain relationship of coal can be categorized into four distinct phases: compaction, elasticity, plasticity, and failure.^39,40^ The numerical model attains equilibrium following its construction, leading to the particle aggregate within the model also reaching equilibrium. As a result, the initial state of the numerical model tends to be dense. Consequently, the stress–strain curves during the early loading stage exhibit only minor fluctuations before transitioning directly into the linear elasticity stage, eventually progressing into the dense stage. As depicted in Fig. 6, it can be observed that the slopes of the three coal types, each representing a distinct level of metamorphism, exhibit a decreasing trend during the elastic phase as the temperature rises. During the yielding stage, as deformation increases, the rate of stress growth lowers. This is seen in the stress–strain curve, where a deviation from linearity occurs. Additionally, new fissures and existing fissures expand and penetrate, leading to irreversible distortion of the coal body structure. During the destructive phase, internal cracks within the coal body propagate, resulting in the formation of visible cracks. As the stress applied to the coal body approaches its ultimate bearing capacity, the coal body experiences significant deformation and a rapid decrease in strength. Consequently, the coal body loses its ability to bear loads, leading to a noticeable residual strength after the peak load is reached. Additionally, the plasticity of the coal body becomes more pronounced, while the occurrence of stress decline gradually diminishes or ceases altogether.

### Strength evolution law

**Figure 7:**
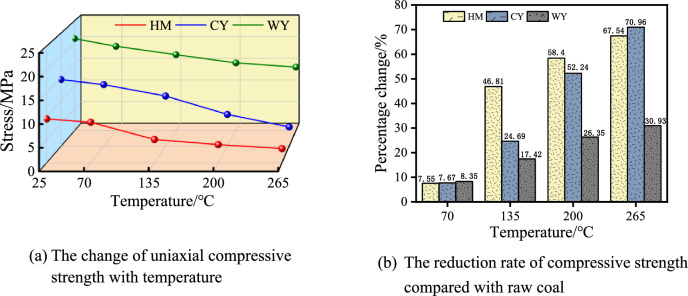
Relationship between compressive strength and oxidation temperature.

The evolution law of uniaxial compressive strength of three coal samples after different temperature treatment is shown in Fig. 7. The stress–strain curve depicted above illustrates notable variations in the strength of different coal kinds following distinct temperature treatments. Moreover, the rates and patterns of these changes also exhibit dissimilarities.

According to the data presented in Fig. 7, it can be observed that the compressive strength of HM exhibited a progressive reduction from 9.40 MPa to 3.06 MPa as the temperature increased. The compressive strength of coal samples decreased by 7.55% when heated from its initial state to a temperature of 70 °C. However, this decrease is not considered statistically significant. This lack of significance can be attributed to the absence of intense thermal expansion at this temperature point, which indicates that the coal body remains relatively stable and its load-bearing capacity does not experience significant weakening. The compressive strength of the coal body exhibited a gradual decrease after undergoing treatment at temperatures of 200 °C and 265 °C. Specifically, the compressive strength decreased by 58.40% and 67.45% respectively, when compared to the original coal. Moreover, the compressive strength of the coal body decreased continuously from 14.74 MPa to 4.28 MPa as the temperature increased from 25 °C to 265 °C. Additionally, it was observed that the compressive strength of the coal body was lower after treatment at temperatures of 70 °C and 135 °C, in comparison to the original coal. Following exposure to temperatures of 70 °C and 135 °C, the compressive strength of the coal underwent reductions of 7.67% and 24.69% respectively. These reductions were found to be statistically significant when compared to the compressive strength of the untreated coal sample. Following the treatment at temperatures ranging from 200 to 265 °C, there was a further drop in compressive strength. Specifically, when compared to the original coal, the compressive strength exhibited a reduction of 52.24% and 70.96% respectively. The decline in compressive strength of coal can be attributed to the heating of its crystalline particles, which causes unequal expansion. This thermal interaction among the particles leads to the breaking of the coal body, ultimately reducing its mechanical qualities^41^. The compressive strength of WY exhibited a negative correlation with rising temperature, but with a gradual decline from 20.95 MPa to 14.47 MPa. Following treatment at temperatures of 70, 135, 200, and 265 °C, the coal exhibited reductions in its properties by 8.35%, 17.42%, 26.35%, and 30.93%, respectively, as compared to its initial state.

Based on the aforementioned analysis, it was observed that the compressive strength of lignite exhibited a notable decline within the temperature range of 70–135 °C. This decline can be attributed to the heightened thermal expansion of lignite during this stage. Additionally, the inadequate capacity of low-rank coal to withstand thermal stress resulted in the formation of numerous thermal cracks. Furthermore, the cementation between coal particles was disrupted, leading to a substantial reduction in uniaxial compressive strength^42^. These findings indicate that the strength of low-rank coal is particularly susceptible to temperature variations. At lower temperatures, middle rank coal exhibits a notable capacity to withstand thermal expansion and thermal stress, resulting in a limited increase in the number of thermal cracks and a moderate decrease in strength. However, when subjected to high temperatures ranging from 200 to 265 °C, the middle rank coal experiences a significant increase in thermal cracks and a substantial reduction in uniaxial compressive strength, measuring only 4.28 MPa. This suggests that while middle rank coal can retain a certain level of strength under low temperature conditions, it is unable to withstand damage caused by high temperatures. After undergoing treatment at a temperature of 265 °C, the high-rank coal exhibited a compressive strength of approximately 15 MPa. Additionally, the occurrence of thermal cracks was very small, indicating that the coal body maintained a relatively stable condition. It can be seen that compared with the other two metamorphic coals, temperature has the least influence on the strength of high rank coal. The findings suggest that as the degree of coal deterioration increases, the influence of temperature on coal strength diminishes progressively.

### Changes in modulus of elasticity

The modulus of elasticity is a measure of the level of difficulty involved in inducing elastic deformation in a given specimen. A higher numerical value signifies more rigidity of the specimen, leading to a correspondingly higher level of stress necessary to induce a certain elastic deformation. In alternative terms, when subjected to a lesser specific parameter, the specimen experiences elastic deformation at a particular stress threshold. The concept of elasticity in the modulus of elasticity pertains to a specimen's capacity to withstand elastic deformation caused by the application of a unit of force, resulting in the desired stress-induced deformation. The index shown herein pertains to the material's capacity to withstand elastic deformation, which is analogous to the stiffness exhibited by a conventional spring.

**Figure 8:**
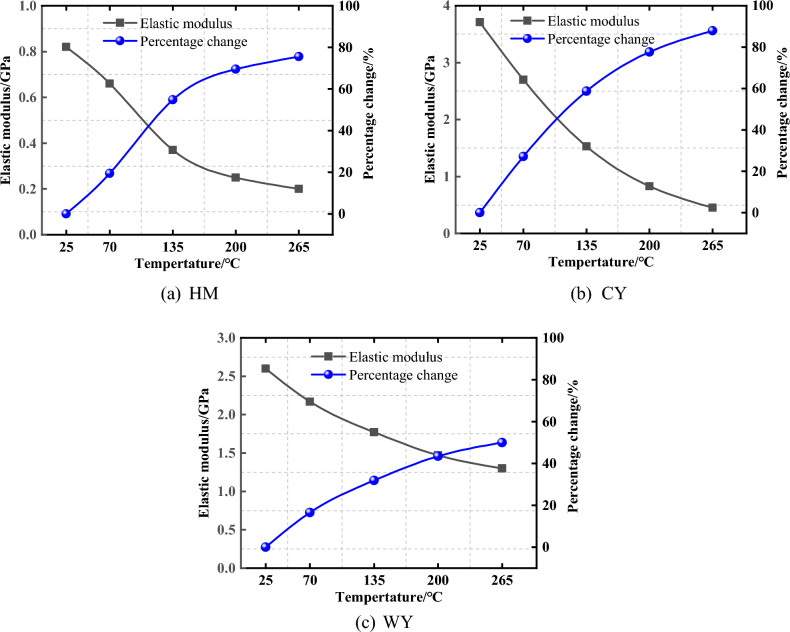
Relationship between modulus of elasticity and oxidation temperature.

Figure 8, illustrates the relationship between temperature and the decrease in the modulus of elasticity of HM coal. Reductions of 19.51% and 54.88% were reported when the initial temperature of the coal was increased to 70 °C and 135 °C, respectively. The period in question witnessed a significant decline. The modulus of elasticity exhibited a gradual deceleration in its fall, reaching 69.51% and 75.61% at temperatures of 200 °C and 265 °C, respectively. In general, there was a reduction in the modulus of elasticity, specifically from 0.82 GPa to 0.20 GPa. The elastic modulus of CY material has seen reductions of 27.22%, 58.76%, 77.63%, and 87.87% after undergoing treatments at temperatures of 70, 135, 200, and 265 °C, respectively. The initial elastic modulus of CY was 3.71 GPa, which has been reduced to 0.45 GPa. A gradual deceleration in the decline of the elasticity modulus was seen with increasing temperature. Following exposure to Celsius treatments, the elasticity modulus experienced a decrease of 16.54%, 31.92%, 43.46%, and 50.00% in comparison to the initial coal sample. This reduction corresponds to a decrease in elasticity modulus from 2.60 GPa to 1.30 GPa. The observed data exhibited a decrease in velocity as the temperature increased.

The data presented indicates a discernible pattern in the modulus of elasticity of low-rank coal, wherein it undergoes a gradual decrease during the initial heating stage before reaching a plateau in the rate of drop. The deformation characteristics of the low-rank coal body exhibit increased sensitivity to variations in temperature, potentially attributed to the creation of thermal cracks during the initial stages of heating. The occurrence of thermal cracks at an early stage within the internal structure of a low-grade coal deposit has the capacity to result in detrimental effects. As the temperature rises, an increased number of thermal cracks become evident, leading to the degradation of the interior structure. Furthermore, it has been observed that a decrease in the elastic modulus of a coal body increases its vulnerability to injury and deformation^43^. After the temperature increases, the curve exhibits a steady deceleration, and the level of damage to the coal body eventually stabilizes. The coal type with the lowest final elastic modulus of 0.2 GPa has the worst resistance to elastic deformation after undergoing heat treatment, indicating that lower-order coals possess a reduced capacity to withstand such deformation. The elastic modulus of middle-order coal had a notable decrease mostly during the warming phase, with a loss of 87.87%. This finding shows that among the three coals, temperature has the greatest weakening effect on the elastic deformation resistance of medium rank coal. The decrease in elastic modulus of high-ranking coal exhibits minimal significance in comparison to the preceding two coal kinds. Moreover, subsequent to the completion of the final treatment process at a temperature of 265 °C, the modulus of elasticity remains constant at 1.3 GPa, exhibiting a noteworthy increase in comparison to coals of intermediate and lower ranks. From this, it can be concluded that higher-ranked coal exhibits the lowest susceptibility to tempera-ture-induced elastic deformation, making it the most resistant to deformation after heat treatment. As the degree of metamorphism increases, the relationship between temperature and the deformation parameters of the coal body becomes less sensitive.

### Strain characteristics

The peak strain is defined as the strain value at which the coal body reaches its compressive strength, serving as the primary indicator for assessing the plasticity of the coal body. The plasticity of the specimen is directly proportional to the magnitude of the peak strain, indicating that a higher peak strain corresponds to increased plasticity. Conversely, a lower peak strain is indicative of greater brittleness, since the specimen exhibits reduced plasticity.

**Figure 9:**
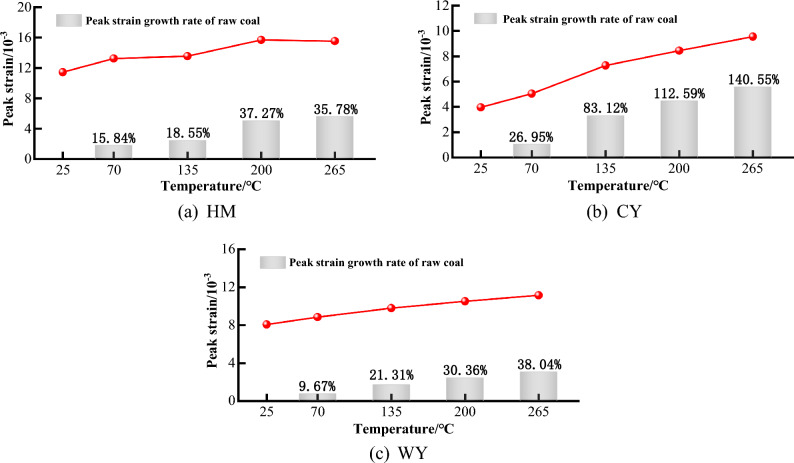
Relationship between peak strain and temperature.

As illustrated in Fig. 9, the maximum strain increased from 11.43 × 10^–3^ to 13.55 × 10^–3^ at temperatures of 25 °C, 70 °C, and 135 °C HM, representing respective increments of 15.84% and 18.55% compared to the initial coal strain. At a temperature of 200 °C, the peak strain exhibited a notable escalation to 15.69 × 10^−3^, signifying a relative rise of 37.27% when compared to the initial coal sample. At this particular temperature, the coal samples exhibited their highest level of brittle toughness, with a peak strain of 15.52 × 10^−3^ seen at 265 °C. This value represents a significant increase of 35% compared to previous measurements. In comparison to the initial coal sample, there was a significant 78% rise in HM peak strain observed at temperatures of 25, 70, 135, 200, and 265 °C. At a temperature of 200 °C, a marginal reduction was observed in comparison. Additionally, there appeared to be a deceleration in the influence of temperature on the brittle toughness of HM. The observed peak strains at temperatures of 135, 200, and 265 °C were 3.97 × 10^−3^, 5.04 × 10^−3^, 7.27 × 10^−3^, 8.44 × 10^–3^, and 8.55 × 10^−3^, respectively. These values indicate an increase of 18.55% compared to the original coal. The coal samples exhibited respective increases of 26.95%, 83.12%, 112.59%, and 140.55%. Furthermore, it was observed that there was a gradual increase in the peak strain of CY as the temperature increased. Furthermore, it was observed that the maximum strain of WY also shown a nearly linear augmentation as the temperature increased, ranging from 8.07 × 10^−3^ to 11.14 × 10^−3^. This corresponds to a 38.04% escalation compared to the initial coal sample.

Upon careful investigation, it becomes apparent that the maximum strain exhibited by the three coal specimens had a significant rise as the temperature was raised. The rise in temperature causes the development of thermal cracks inside the internal pore structure of the coal body, leading to the deterioration of its integrity and consequently prolonging the compaction process. According to the reference cited^44^, the coal samples demonstrate plastic behavior when subjected to heating, suggesting that the temperature has an impact on the plasticity of the coal specimens. The strain growth rate of HM at high temperature decreases, indicating that the effect of high temperature treatment on the plasticity of low-rank coal decreases. Because as the temperature gradually increases, the compressive strength and elastic modulus of HM are in a very low state, and the thermal cracks increase greatly, resulting in a rapid failure after loading, so that the peak strain does not increase much. The coal type denoted as CY demonstrates the most significant alteration in strain when compared to the other two coal types that were examined. The observed phenomenon can be ascribed to the rise in temperature, leading to changes in the compressive strength and modulus of elasticity of CY. These alterations facilitate the occurrence of thermal cracks in coal, resulting in destructive consequences. Furthermore, the swift decline in modulus of elasticity results in a loss in the ability of CY to withstand deformation. The maximal strain of WY exhibits a linear rise. This phenomenon can be attributed to the consistent and low occurrence of thermal cracks in WY across various temperature points. Furthermore, the internal structural integrity of WY exhibits a notable degree of strength, hence exerting a limited influence on its plasticity. Hence, the influence of temperature on the flexibility of high-order coal is quite minimal. Different temperatures cause the early pores and fissures to display varied degrees of development and expansion. The interconnected fractures contribute to the progressive deterioration of the internal structure of the coal body and a subsequent decrease in its mechanical characteristics, ultimately leading to its collapse. Several researchers have also noted the adverse effects of temperature on the load-bearing capacity and deformation characteristics of coal samples. It has been shown that the strength and resistance to deformation of coal samples decrease as the heating temperature increases. The application of heat triggers a sequence of structural transformations in coal molecules, ultimately leading to the disruption of intermolecular forces^45^.

### Failure modes

Following the specimen damage, a clear main crack appears on the exterior, and there exist damages and cracks internally. Nevertheless, the PFC2D software draws upon a two-dimensional simulation that can reproduce damage patterns but not the three-dimensional propagation of such cracks. As a result, disparities may exist in the main crack's location when compared to experimental outcomes. Edge chipping could potentially result from internal damage in the simulated sample or from the excessive strength of the ballasted wall.

**Figure 10:**
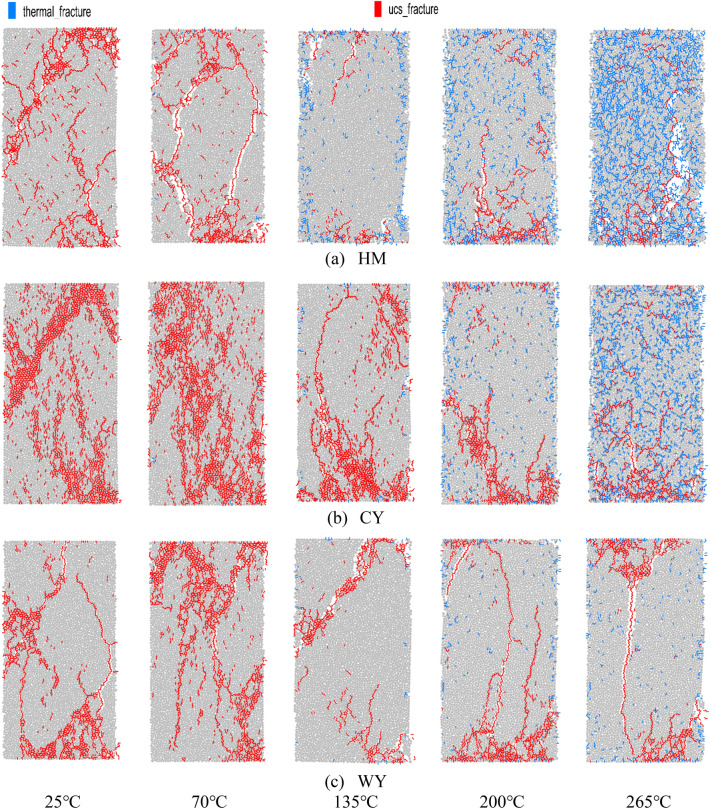
Loading damage crack distribution.

Figure 10, depicts the distribution of loading-induced cracks, as illustrated in Fig. 10a. The low-order coal body primarily exhibits shear damage at lower temperatures. However, as the temperature rises, the coal body undergoes split damage, influenced by the presence of thermal cracks in its vicinity. The integrity of the coal body diminishes progressively as the temperature rises. Although compression cracks are not significantly prevalent during the destabilization and damage of the coal sample as a whole, the collapse primarily occurs due to the development of thermal cracks surrounding the thermal cracks. These thermal cracks cause a certain level of damage to the load carrying capacity of the coal body before they become loaded. At a temperature of 265 °C, the treatment of coal samples mostly results in the formation of a fracture network and a decrease in integrity, leading to their annihilation. The presence of a higher quantity of fragmented particles on the surface can be attributed to the coal body containing a significant number of thermal cracks. As a result, the bonding between particles is weakened, leading to the formation of a greater number of pores. The outcome of this phenomenon is an increased likelihood of the occurrence of microcracks and secondary rupture surfaces during the loading process. These microcracks and secondary rupture surfaces have the effect of diminishing the bearing capacity of the coal, weakening its resistance to deformation, and intensifying the degree of fragmentation during destruction. Additionally, the presence of a greater number of thermal cracks further complicates the process of coal body destruction. The coal located in the middle-order, as depicted in Fig. 10b, primarily undergoes shear-induced damage. As the temperature rises, the occurrence of thermal cracks becomes more widespread throughout the coal body. Consequently, the mode of damage gradually shifts, with the cracks expanding and giving rise to a tensile damage surface. This surface extends from the upper to the lower ends of the specimen. The combined damage mechanism involves both axial cleavage damage and shear damage, ultimately leading to the collapse of the coal specimen. Thermal cracks, which are prevalent, are commonly addressed with a treatment involving exposure to a temperature of 265 °C. The resulting damage caused by external forces is primarily focused in the lower region. The focus is primarily centered on the lower portion. According to the findings presented in Fig. 10c, the predominant damage mode observed in high-order coal is characterized by split damage, with a relatively low occurrence of thermal cracks distributed across the coal body. At a temperature of 200 °C, thermal cracks were observed at both the upper and lower ends of the coal samples. These thermal cracks initiated the destruction of compression cracks, which gradually propagated from the lower end of the thermal cracks, resulting in the formation of a macroscopic split damage surface. The coal samples experienced a loss of structural integrity due to uniaxial compression at a temperature of 265 °C. This resulted in the formation of cracks at both the upper and lower ends of the loaded samples, which subsequently extended across the samples, causing axial splitting damage. The observation reveals that elevated temperatures result in an increased occurrence of cracks within the sample, accompanied with interstitial penetration. Consequently, the fracture behavior of the specimen after the application of external forces is influenced by the presence of cracks induced by elevated temperatures. As the temperature increases, the damage pattern of the loaded coal body becomes more intricate and the integrity of the coal body deteriorates. As the level of metamorphism escalates, the influence of temperature on the coal's damage pattern rapidly diminishes.

The analysis of the impact of temperature on coal damage effect at a microscopic level can be explained as follows: As the temperature increases, the particles in the coal body undergo thermal expansion, causing the fine structure of the coal body to be disrupted. Consequently, the bonding area between particles gradually diminishes, resulting in a loosening of the fine structure. Additionally, the expansion and deformation caused by thermal stress further weakens the bond between particles and alters the fine structure of the coal body. This progressive deterioration of cohesion leads to a gradual decrease in the bonding area between particles when subjected to external loads. Ultimately, this reduction in cohesion contributes to the overall decrease in the coal body's structural integrity. The application of external load can induce stress concentration in regions where the particle bonding area is limited and the degradation of the fine structure is pronounced. This, in turn, facilitates the occurrence of microcracks and secondary rupture surfaces during the loading of rock specimens. Consequently, the bearing capacity of coal is diminished, and the damage pattern becomes more intricate, gradually compromising the integrity of the coal.

## Conclusion

Taking the particle discrete element method as the theoretical basis, a parallel bonded contact model was adopted as the compressed material, and the parameters of the PFC model were calibrated, so that the constructed PFC model has the same macroscopic mechanical properties as the actual oxidized coal specimen. On this basis, the uniaxial compression experiments at different characteristic temperatures were simulated by adding the thermal module, and the numerical results coincided with the results of previous indoor experiments^8,28,29^, which proved the reliability of the simulation results. The main conclusions of this paper into the following:

1. The occurrence and propagation of cracks are observed to be positively correlated with rising temperatures. Notably, low-rank coal exhibits the earliest manifestation of thermal cracks, accompanied by the highest crack propagation rate. During the initial phase of temperature increase, middle rank coal exhibits a relatively stable state. However, when exposed to high temperatures and experiencing more pronounced thermal expansion, it undergoes a significant number of thermal cracks. In contrast, high rank coal maintains a relatively stable state and exhibits the lowest occurrence of thermal cracks. The thermal expansion resistance of coal increases with greater coal rank, resulting in reduced temperature-induced effects on the macroscopic structure.
2. Through the analysis of stress–strain curve, it can be seen that with the increase of temperature, the compressive strength and elastic modulus of coal decrease, the anti-deformation ability decreases, the peak strain increases, and the plasticity continues to increase, indicating that the damage degree of hot cracks to coal is deepening. With the increase of temperature, the peak strength of the three kinds of coal decreases, the linear elastic slope decreases, the peak strain increases, and the residual strength increases.
3. Upon doing a more comprehensive examination of mechanical properties, including the stress–strain curve, compressive strength, and elastic modulus, it becomes evident that the impact of temperature on the mechanical properties of coals varies significantly depending on the degree of metamorphism. Low-rank coal experiences notable thermal degradation at lower temperatures, resulting in a substantial reduction in its compressive strength. Specifically, at 135 °C, the compressive strength reduces by 46.81%, a significantly higher decline compared to the other two coal categories. Furthermore, at 265 °C, the compressive strength of low-rank coal is merely 3.06 MPa. Elevated temperatures expedite the thermal degradation of low-rank coal, hence leading to a subsequent decrease in its mechanical properties, namely strength and stiffness. The mechanical properties of middle and low rank coals exhibited significant alterations following heat treatment at a temperature of 265°C. Notably, the strain increased by 140.55%, surpassing that of the other two ranks. Conversely, the high rank coals displayed considerably lower mechanical properties compared to the middle and low rank coals following the identical temperature treatment. As the degree of metamorphism increases, the influence of temperature on the mechanical properties of coal diminishes progressively.
4. The examination of the damage pattern reveals that the elevated temperature altered the fracture pattern of the specimens, mostly influenced by the cracks induced by the high temperature. Low-rank coal exhibits a higher prevalence of thermal fractures compared to other coal ranks. It encompasses a spectrum of damage, ranging from shear damage to a combination of shear and cleavage composite damage. This results in a more intricate pattern of damage and the least favorable structural integrity following loading. Higher-order coal exhibits a reduced occurrence of thermal cracks and demonstrates greater stability, resulting in less impact on the damage mode.

## Data Availability

The data used to support the findings of this study are available from the corresponding author upon request.
